# Evidence of Renal Iron Accumulation in a Male Mouse Model of Lupus

**DOI:** 10.3389/fmed.2020.00516

**Published:** 2020-09-08

**Authors:** Lindsey R. Theut, Del L. Dsouza, Ryan C. Grove, Erika I. Boesen

**Affiliations:** Department of Cellular & Integrative Physiology, University of Nebraska Medical Center, Omaha, NE, United States

**Keywords:** kidney, iron, lupus, transferrin, urine

## Abstract

Lupus nephritis represents a common and serious complication of the autoimmune disease Systemic Lupus Erythematosus (SLE). Clinical studies suggest that several proteins related to iron metabolism, including transferrin, serve as urinary biomarkers of lupus nephritis. We previously reported that in female NZBWF1 mice, a commonly used mouse model of SLE with a female sex bias, increased urinary transferrin excretion and renal iron accumulation occur around the onset of albuminuria. The current study investigated whether similar findings occur in male mice of a different mouse model of SLE, the MRL/*lpr* mouse. Two different cohorts were studied: MRL/*lpr* mice at an early, pre-albuminuric age (8 weeks), and after developing albuminuria (>100 mg/dL, confirmed by ELISA); age-matched MRL/MpJ control strain mice served for comparison. Urinary transferrin excretion was dramatically increased in the older, albuminuric MRL/*lpr* mice compared to the age-matched MRL/MpJ (*P* < 0.05), but there was no significant difference between strains at 8 weeks of age. Similarly, there were no significant differences between strains in renal cortical or outer medullary non-heme iron concentrations at 8 weeks. In the older, albuminuric MRL/*lpr* mice, renal cortical and outer medullary non-heme iron concentrations were significantly increased compared with age-matched MRL/MpJ mice, as was the expression of the iron storage protein ferritin (*P* < 0.01). Together, these data show that increased urinary transferrin excretion and renal tissue iron accumulation also occurs in albuminuric male MRL/*lpr* mice, suggesting that renal iron accumulation may be a feature of multiple mouse models of SLE.

## Introduction

The autoimmune disease Systemic Lupus Erythematosus (SLE) affects multiple organ systems in the body, and inflammation and damage to the kidneys (lupus nephritis) is a common and serious complication of SLE. Indeed, renal function is impaired in ~40% of lupus nephritis patients, increasing mortality risk; progression to end-stage kidney disease occurs in a significant number of patients (1, 2). The mechanisms of renal injury in SLE include initiation of inflammation via immune complex deposition, changes in the production of locally-acting factors such as vascular endothelial growth factor, endothelin-1, cytokines, and pro-fibrotic factors, as well as alterations in cellular oxygenation and metabolism (1).

While much of the current clinical therapeutic armamentarium for SLE rightly focuses on combating the aberrant immune response and inflammation seen in this autoimmune disease, identification of non-immune mechanisms that contribute to the progression of renal injury in SLE may provide novel complementary therapeutic approaches. In this vein, multiple proteins involved in iron metabolism and handling have been identified as plasma or urinary biomarkers of lupus nephritis and disease flares, including the iron carrier protein transferrin, neutrophil gelatinase-associated lipocalin (NGAL), the ferroxidase enzyme ceruloplasmin, the regulatory protein hepcidin, and the iron storage protein ferritin (3–7). Iron is an important redox-active component of multiple enzymes and metalloproteins in the body. However, these properties of iron also create the potential of unbound or “labile” iron to promote cellular damage through induction of oxidative stress, lysosomal and mitochondrial damage, endoplasmic reticulum stress and cell death via ferroptosis (8–11). An influence of iron on the immune system [reviewed recently by Cronin et al. (12)] may also come into play in the context of SLE. Indeed, a recent study reported increased iron levels in CD4^+^ T cells of SLE patients which was associated with epigenetic changes leading to the upregulation of various genes related to autoimmunity (13). As such, dysregulation of iron homeostasis could contribute to kidney injury via a number of mechanisms. Consistent with dysregulation of iron homeostasis in SLE, we previously reported increased iron accumulation in the kidneys of female (New Zealand Black × New Zealand White) F1 (NZBWF1) mice compared to age-matched female New Zealand White (NZW) mice near the onset of albuminuria (14). This well-established polygenic model of SLE is created by crossing the New Zealand Black (NZB) mouse, which itself displays an autoimmune-mediated hemolytic anemia and an SLE-like syndrome including anti-nuclear auto-antibody production and glomerulonephritis in a small percentage of animals, with the NZW mouse, which greatly increases the prevalence of features of SLE in the F1 offspring, including autoimmunity and development of immune complex-mediated glomerulonephritis (15, 16). The increased renal iron accumulation observed previously in the NZBWF1 model was accompanied by increased urinary excretion of transferrin in albuminuric NZBWF1 mice (14), echoing biomarker data reported by others in SLE patients (5, 7, 17, 18). Similar to SLE patients, approximately 90% of whom are female (19), the NZBWF1 mouse model demonstrates a female sex bias [reviewed in (15)]. As reviewed recently (20), male SLE patients often have a more aggressive and rapidly progressing clinical course, including a higher incidence of lupus nephritis, highlighting the relevance of studying SLE disease mechanisms in males as well as females.

Given the heterogeneity of disease manifestations in SLE patients, it is important to evaluate disease mechanisms and pathological findings in multiple mouse models of SLE. Another mouse model of SLE with prominent development of glomerulonephritis is the Murphy Roth's (or Recombinant) Large (MRL)/lymphoproliferation (*lpr*) mouse model. The *lpr* mutation was discovered to be a mutation in the *Fas* (or CD95) gene, and dramatically accelerates the development of the autoimmune phenotype seen with the MRL genetic background (15). A recent study by Scindia and colleagues (21) reported histological evidence of iron deposition within the kidney of female MRL/*lpr* mice although the amounts were not quantified, and that treatment of mice with hepcidin ameliorated renal injury. To continue to expand knowledge regarding whether renal iron accumulation occurs more broadly in mouse models of SLE including in males, the current study tested whether renal iron accumulation quantified by our previously used method as well as increased urinary transferrin excretion also occurs in male MRL/*lpr* mice. Male MRL/MpJ mice were used as age-matched controls in the current study.

## Materials and Methods

### Animal Procedures

All procedures were approved in advance by the University of Nebraska Medical Center Institutional Animal Care and Use Committee and were in agreement with the National Institutes of Health's Guide for the Care and Use of Laboratory Animals. Male MRL/*lpr* and MRL/MpJ mice were purchased at age 7 weeks from Jackson Laboratories (Bar Harbor, ME) and group housed four per cage in our animal facility on a 12-h light/dark cycle with *ad libitum* access to a standard diet (Teklad LM-485 mouse/rat sterilizable diet, Envigo; the iron content is 240 mg/kg according to the manufacturer's specification sheet) and drinking water. Commencing at 8 weeks of age, mice were placed in metabolic cages for 24 h for urine collection, with free access to food and water. Mice were then either euthanized (isoflurane overdose; Piramal Enterprises Ltd., Digwal Village, India) for blood and tissue collection, or underwent additional 24 h urine collections weekly to monitor for the onset of albuminuria using Albustix® colorimetric dipsticks (Siemens Healthcare Diagnostics, Tarrytown, NY), with albuminuria defined as a reading of at least 2+ to 3+ on a 0–4 scale, representing ≥ 100–300 mg/dL, respectively. At that point, mice were euthanized for blood and tissue collection. Urinary albumin excretion was then accurately determined by ELISA (Albuwell M, Exocell, Philadelphia, PA), and albuminuric mice with confirmed excretion of >100 mg/dL were included in the study along with their contemporaneously collected MRL/MpJ control counterpart to ensure age-matching (median of 18 weeks, range 15–22 weeks).

### Tissue Collection and Sample Processing

Blood was centrifuged at 10,000 g for 5 min at 4°C and plasma collected for determination of creatinine, blood urea nitrogen (BUN), and iron using QuantiChrom Creatinine, Urea, and Iron Assay kits, respectively (BioAssay Systems, Hayward, CA). Plasma hepcidin (hepcidin-1) was measured by ELISA (Hepcidin-Murine Compete^TM^ ELISA, Intrinsic Life Sciences, La Jolla, CA). Hematocrit was determined by standard procedures following the centrifugation of a small blood sample in a heparinized mylar® wrapped capillary tube (Drummond Scientific Company, Broomall, PA). Plasma auto-antibodies were measured using a mouse anti-double stranded DNA (anti-ds DNA) IgG-specific ELISA kit (ADI, San Antonio, TX).

Spleen and whole kidney weights were recorded, and then small samples of liver, renal cortex, and outer medullary tissue were rapidly dissected, frozen in liquid nitrogen and stored at −80°C. Tissue non-heme iron concentrations were determined by microplate colorimetric assay following acid extraction and reaction with bathophenanthroline reagent, as described by Grundy et al. (22) and performed previously in our lab (14). Renal cortex and outer medullary tissue ferritin heavy chain (H-ferritin) levels were compared between groups by western blotting. Briefly, the tissues were homogenized in RIPA buffer with protease inhibitors [final concentrations: 1 mM phenylmethylsulfonyl fluoride, 2 μM Leupeptin, 1 μM Pepstatin A, a 1:1000 dilution of 0.1% Aprotinin, 14.3 mM 2-mercaptoethanol (all from Sigma-Aldrich, St. Louis, MO)]. Following centrifugation at 10,000 g for 5 min, supernatants were collected, and protein concentrations measured by the bicinchoninic acid (BCA) method (Pierce BCA Protein Assay kit, Thermo Fisher Scientific, Rockford, IL). The proteins were then solubilized in Laemmli sample buffer and separated by electrophoresis on 4–15% SDS-PAGE gels (Bio-Rad Laboratories Inc., Hercules, CA). After transfer to polyvinylidene difluoride (PVDF; Immobilon-FL, Merck Millipore, Cork, Ireland), total protein staining was performed according to the manufacturer's instructions (REVERT, Li-Cor Biosciences, Lincoln, NE), membranes were blocked for 1 h (50:50 Li-Cor Blocking Buffer and Tris-buffered saline) and then incubated overnight at 4°C with a rabbit monoclonal primary antibody against ferritin (1:1,000, ab75973, Abcam, Cambridge, UK, which recognizes ferritin heavy chain in mouse tissue) followed by a goat anti-rabbit 790 nm infrared dye-conjugated secondary antibody for 1 h at room temperature (1:10,000, Life Technologies, Grand Island, NY). Immunoreactivity was then visualized and quantified using an Odyssey Infrared Imaging System (Li-Cor Biosciences), allowing for two-color detection, and Image Studio Lite (v. 5.2; Li-Cor Biosciences) was used to quantify the detected signals. Total protein staining was used as the loading control, and the ratio of the protein of interest to total protein stain was normalized to the average of the MRL/MpJ samples within each blot.

The contralateral kidneys were immersion-fixed in 10% neutral buffered formalin for 24 h and then paraffin-embedded. Kidney sections were stained with Periodic-Acid Schiff (PAS; 3 μm sections) and Perl's Prussian blue with nuclear fast red counterstain (4 μm sections) by the Tissue Sciences Facility histology core at the University of Nebraska Medical Center using standard methodology. PAS-stained sections were examined qualitatively for evidence of histological injury, with 10 randomly-chosen 20× images of the renal cortex evaluated per mouse. For Perl's Prussian blue stained sections, areas of positivity are described below.

### Statistical Analysis

Data are presented as mean ± SEM for n of 8 mice per group for the 8-week-old mice and 7 per group for the older mice, except as indicated due to insufficient tissue or plasma for analysis. Statistical analysis was performed using GraphPad Prism (version 6.01 for Windows, GraphPad Software, La Jolla, CA). Comparisons between age-matched groups are by Student's unpaired *t*-test or Mann–Whitney *U*-test if *F* test revealed a significant difference in variance between groups. *P* < 0.05 was considered statistically significant.

## Results

### Urinary Markers and Disease Parameters in Male MRL/*lpr* Compared to MRL/MpJ Mice

**Figure 1:**
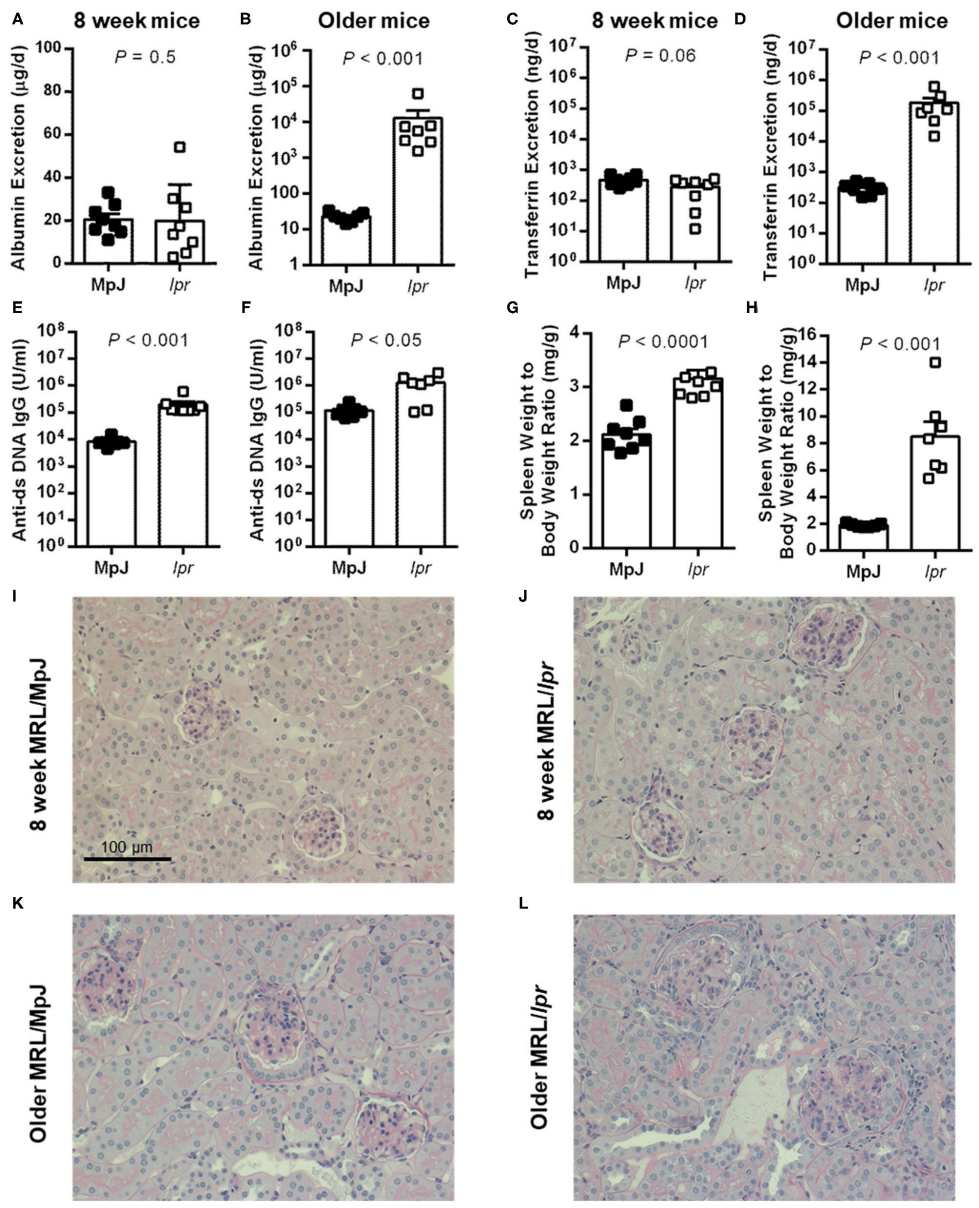
Urinary markers and disease parameters in male MRL mice. **(A,C,E,G,I,J)** depict data from 8-week-old mice (*n* = 8 per group except for plasma anti-ds DNA IgG where *n* = 7 for MRL/MpJ). **(B,D,F,H,K,L)** depict data from albuminuric MRL/*lpr* mice and their age-matched MRL/MpJ counterparts (*n* = 7 per group; median of 18 weeks, range 15–22 weeks). Rates of urinary albumin excretion **(A,B)** and transferrin excretion **(C,D)** were determined following 24 h metabolic cage collections of urine. Also shown are plasma anti-ds DNA IgG autoantibody concentrations **(E,F)** and spleen weight to body weight ratio **(G,H)**. Data are presented as individual data points as well as mean ± SEM for each group. *P*-values were determined by Student's unpaired *t*-test **(C,G)** or Mann–Whitney *U*-test when *F* test revealed a significant difference in variance between groups (all other panels). **(I,J)** Show representative cortical images of PAS-stained kidney sections from 8-week-old MRL/MpJ **(I)** and MRL/*lpr* mice **(J)**, and from older MRL/MpJ **(K)** and MRL/*lpr* mice **(L)**.

Urinary albumin excretion was low and not significantly different between MRL/MpJ and MRL/*lpr* mice at 8 weeks of age (*P* = 0.5; Figure 1A). Per the experimental design, the older group of MRL/*lpr* mice displayed significantly higher urinary albumin excretion compared to age-matched MRL/MpJ mice (*P* < 0.001; Figure 1B). Similarly, urinary transferrin excretion was not significantly different between the two groups of 8-week-old mice (*P* = 0.06; Figure 1C) but was dramatically elevated in the older MRL/*lpr* mice (1.84 x 10^5^ ± 7.9 x 10^4^ ng/d) compared to MRL/MpJ mice (310 ± 49 ng/d; *P* < 0.001; Figure 1D). Both MRL strains displayed the presence of anti-ds DNA IgG in plasma, with MRL/*lpr* mice having higher plasma anti-ds DNA IgG concentrations than MRL/MpJ at both 8 weeks and in the older cohort (Figures 1E,F). Similarly, spleen weight to body weight ratios were significantly higher in MRL/*lpr* compared to MRL/MpJ mice in both age groups (*P* < 0.001; Figures 1G,H), despite similar body weights within each age group (37.2 ± 0.7 vs. 38.6 g for MRL/*lpr* and MRL/MpJ mice, respectively at 8 weeks, *P* = 0.08; 44.4 ± 1.3 vs. 42.7 ± 0.7 g in the older groups, *P* = 0.3). Representative images of PAS-stained kidney sections are shown in Figures 1I–L. Glomeruli of 8-week-old mice predominantly appeared normal (Figures 1I,J). Glomeruli of older mice displayed a variable degree of histological injury, qualitatively ranging from mild to moderate for MRL/MpJ (Figure 1K) and moderate to severe in MRL/*lpr* mice (Figure 1L). Mild mesangial expansion and hypercellularity were present in most glomeruli from the MRL/MpJ mice. Severe mesangial expansion, hypercellularity, and loss of capillary lumen were evident in glomeruli of the older MRL/*lpr* mice, consistent with the development of glomerulonephritis. Evidence of tubular injury was also present to a variable degree in older MRL/*lpr* mice, with the presence of casts, hypertrophic, atrophic or dilated tubules, as well as interstitial hypercellularity, likely indicating the presence of infiltrating immune cells.

There was no significant difference in plasma creatinine concentration between MRL/MpJ and MRL/*lpr* at either 8 weeks (0.16 ± 0.04 vs. 0.16 ± 0.02 mg/dL; *P* = 0.9) or older ages (0.24 ± 0.02 vs. 0.27 ± 0.02; *P* = 0.4), indicating that the MRL/*lpr* mice had not yet developed renal failure at the ages studied (15–22 weeks). BUN was mildly increased in MRL/*lpr* mice compared to MRL/MpJ mice at 8 weeks (29 ± 4 vs. 21 ± 1 mg/dL; *P* = 0.051) and further increased in the older cohort (42 ± 5 vs. 23 ± 2 mg/dL; *P* < 0.01). Hematocrit was similar in 8-week-old MRL/MpJ and MRL/*lpr* mice (52.5 ± 0.4 vs. 51.0 ± 0.8% *P* = 0.1; *n* = 7 per group due to lack of successful blood collection from one MRL/MpJ mouse and capillary tube failure for one MRL/*lpr* mouse). Hematocrit was also similar in the older MRL/MpJ and MRL/*lpr* mice (48.0 ± 1.9 vs. 46.3 ± 1.8%; *P* = 0.5). Plasma iron concentrations were not significantly different between 8-week-old MRL/MpJ and MRL/*lpr* mice (353 ± 37 vs. 314 ± 14 μg/dL; *P* = 0.3) or between the older MRL/MpJ and MRL/*lpr* mice (291 ± 33 vs. 338 ± 26 μg/dL; *P* = 0.3). Plasma hepcidin concentration was not significantly different between 8-week-old MRL/MpJ and MRL/*lpr* mice (153 ± 12 vs. 189 ± 18 ng/mL; *P* = 0.1). Hepcidin concentration was variable in the older MRL/MpJ mice (244 ± 33 ng/mL, range of 156–400 ng/mL) but not significantly different to the MRL/*lpr* mice (168 ± 15 ng/mL; *P* = 0.06).

### Renal Iron Accumulation in Male MRL/*lpr* Compared to MRL/MpJ Mice

**Figure 2:**
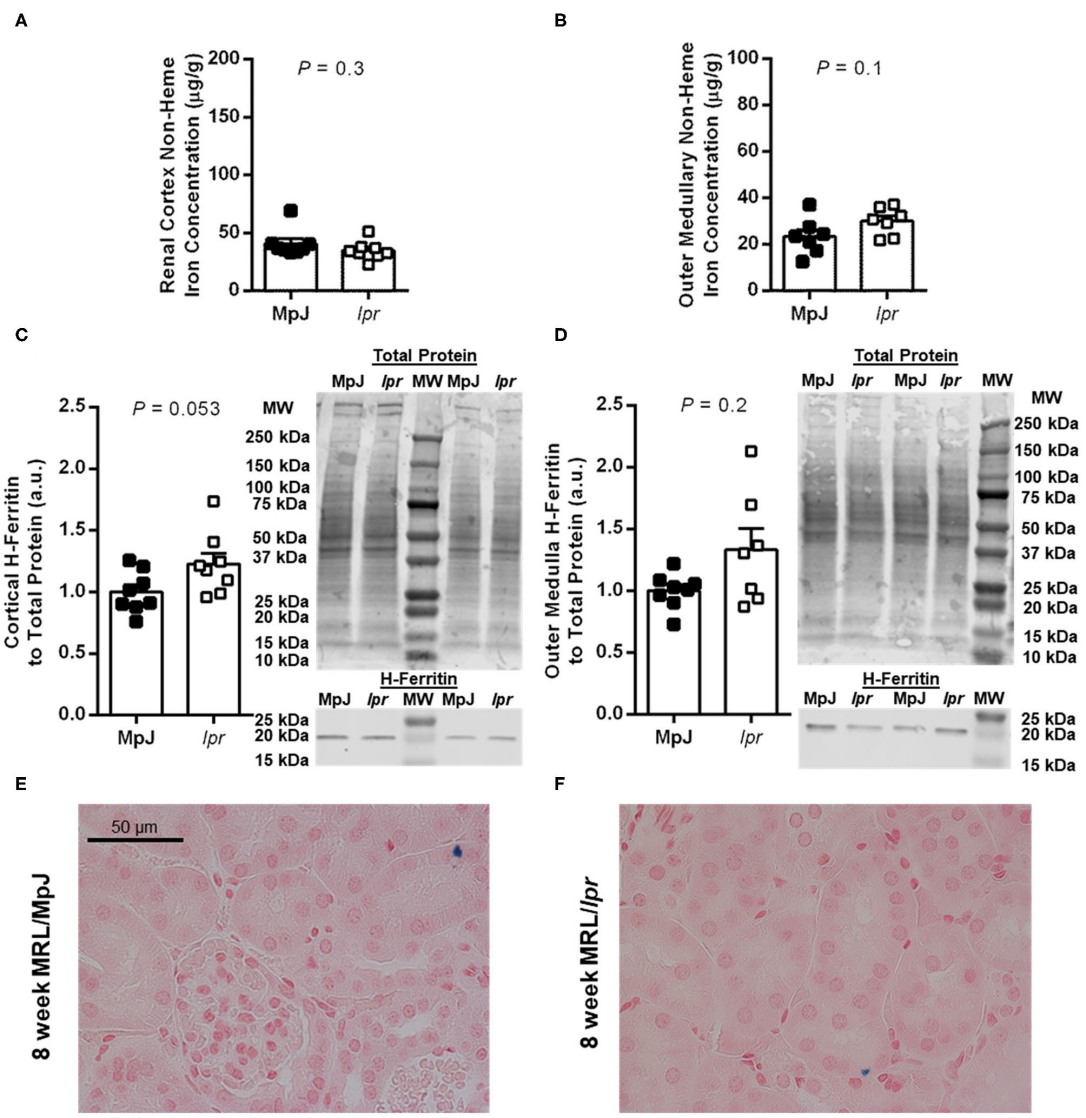
Measures of renal iron accumulation in 8-week-old male MRL mice. Renal tissue non-heme iron concentrations, expressed per g of wet tissue weight, were measured separately in renal cortex **(A)** and outer medulla **(B)** of MRL/MpJ and MRL/*lpr* mice. Western blot analysis of ferritin heavy chain (H-ferritin) was performed on renal cortical tissue **(C)** and outer medullary tissue **(D)**, with the densitometric ratio of H-ferritin to total protein staining presented as arbitrary units (a.u.) after normalizing to the average of the MRL/MpJ group within each blot. Representative images of H-ferritin staining are shown (MW, molecular weight markers), with total protein staining of the same lanes shown above (please note that the difference in intensity of the molecular weight markers between images is due to the two scans being performed at different times and to the total protein stain highlighting the markers). Data are presented as individual data points as well as mean ± SEM for each group, with *n* = 8 per group except for **(B)** where one sample was missing per group, and **(D)** where one sample was missing from the MRL/*lpr* group. *P-*values were determined by Student's unpaired *t*-test **(A–C)** or Mann-Whitney *U*-test **(D)**. **(E,F)** Show areas of renal cortex from kidney sections stained with Perl's Prussian blue [blue pigment; MRL/MpJ, **(E)**; MRL/*lpr*, **(F)**].

Renal tissue non-heme iron concentrations and expression of the iron storage protein H-ferritin were measured separately in the renal cortex and outer medulla. At 8 weeks of age, there were no significant differences between strains in either cortical (*P* = 0.3; Figure 2A) or outer medullary non-heme iron concentrations (*P* = 0.1; Figure 2B; *n* = 7 per group due to insufficient tissue from one mouse per group). There was a trend toward increased renal cortical H-ferritin expression in MRL/*lpr* compared to MRL/MpJ mice, but this did not reach statistical significance (*P* = 0.053; Figure 2C). Outer medullary H-ferritin expression was also not significantly different between the groups of 8-week-old mice (*P* = 0.2; Figure 2D). Essentially no positive Perl's Prussian blue staining was observed in sections of 8-week-old mice from either group, other than a handful of isolated occurrences of an apparent intracellular inclusion as shown in Figures 2E,F.

**Figure 3:**
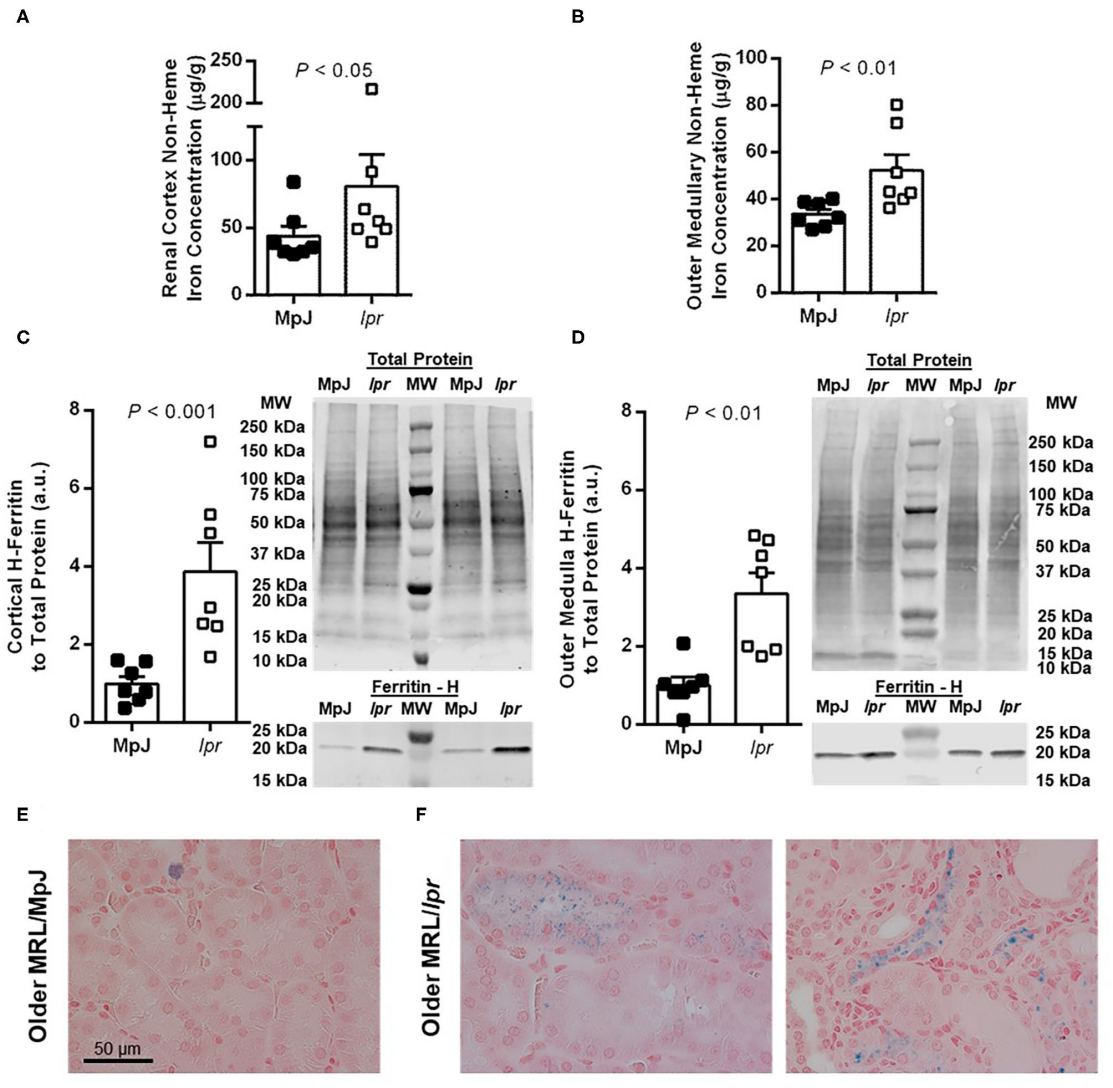
Measures of renal iron accumulation in albuminuric male MRL/*lpr* mice compared with age-matched MRL/MpJ mice (median of 18 weeks, range 15–22 weeks). Renal tissue non-heme iron concentrations, expressed per g of wet tissue weight, were measured separately in renal cortex **(A)** and outer medulla **(B)**. Western blot analysis of H-ferritin was performed on renal cortical tissue **(C)** and outer medullary tissue **(D)**, with the densitometric ratio of H-ferritin to total protein staining presented as arbitrary units (a.u.) after normalizing to the average of the MRL/MpJ group within each blot. Representative images of H-ferritin staining are shown (MW, molecular weight markers), with total protein staining of the same lanes shown above. Data are presented as individual data points as well as mean ± SEM for each group, with *n* = 7 per group. *P*-values were determined by Mann–Whitney *U*-test **(A–C)** or Student's unpaired *t*-test **(D)**. Images shown in **(E,F)** are of Perl's Prussian blue-stained kidney sections from older MRL/MpJ **(E)** and MRL/*lpr* mice **(F)**.

In the older mice, renal tissue non-heme iron concentration was significantly increased in both the renal cortex (*P* < 0.05; Figure 3A) and outer medulla (*P* < 0.01; Figure 3B) of MRL/*lpr* compared with MRL/MpJ mice. Consistent with elevations in tissue iron levels, renal cortical H-ferritin expression was significantly increased in MRL/*lpr* compared to MRL/MpJ mice (*P* < 0.001; Figure 3C), as was outer medullary H-ferritin expression (*P* < 0.01; Figure 3D). Positive Perl's Prussian blue staining was scarce in the older MRL/MpJ mice and was mostly limited to what appeared to be cellular debris or possible macrophages (example given in Figure 3E). Older MRL/*lpr* mice showed focal areas of positive staining in amounts qualitatively consistent with the level of non-heme iron measured in the contralateral kidney. Patterns of staining included puncta in proximal tubular and other tubular cells, some of which appeared atrophic (examples shown in Figure 3F), as well as regions of distorted tubulointerstitial architecture, discrete areas of staining close to clusters of immune cells, and occasional cells that are possible macrophages, similar to Figure 3E. Glomeruli were devoid of positive Perl's staining in all groups (not shown). Average kidney weight-to-body weight ratios for 8-week-old MRL/MpJ and MRL/*lpr* mice were not significantly different between groups (8.0 ± 0.1 vs. 7.7 ± 0.2 mg/g; *P* = 0.2), nor were they significantly different between older MRL/MpJ and MRL/*lpr* mice (8.4 ± 0.3 vs. 9.4 ± 0.6 mg/g; *P* = 0.4). There was no significant difference in liver non-heme iron concentration between 8-week-old MRL/MpJ (171 ± 19 μg/g) and MRL/*lpr* mice (199 ± 20 μg/g; *P* = 0.3). In the older cohorts, liver non-heme iron concentrations were 243 ± 13 μg/g in MRL/MpJ mice and 203 ± 44 μg/g in MRL/*lpr* mice (*P* = 0.053).

## Discussion

Herein we report evidence of increased urinary transferrin excretion and renal iron accumulation in albuminuric male MRL/*lpr* mice compared to age-matched MRL/MpJ control mice. The MRL/*lpr* model significantly affects male as well as female mice, providing a tractable model for the study of SLE manifestations in male mice (15). These findings build on similar observations previously made in female NZBWF1 mice, which displayed increased renal cortical and outer medullary non-heme iron and increased tubular ferritin expression compared with age-matched female NZW control mice (14). Perl's Prussian blue staining, which detects iron deposits typically in the form of hemosiderin, typically reveals no positive staining of normal kidney tissue, which contains much lower levels of iron (in this study, ~20–40 μg/g of non-heme iron in the 8-week-old mice) than tissues normally involved in iron storage and metabolism such as the liver (~150–200 μg/g). Our finding of positive Perl's Prussian blue staining in the older male MRL/*lpr* mice is consistent with a recent report of positive Perl's Prussian blue staining in tubular cells and interstitial areas of 18-week-old female MRL/*lpr* mice (21). Together, these data suggest that increased exposure of the kidney to iron and iron accumulation may be a common feature of mouse models of SLE. In a clinical report (23), iron deposition was observed in proximal tubules of patients with several forms of glomerulonephritis, interstitial fibrosis, or diabetic nephropathy. Whether renal iron deposition also occurs in lupus nephritis patients is unknown, although as discussed above, urinary biomarker studies (3–7) suggest that the kidney is exposed to abnormal levels of proteins related to iron metabolism, including transferrin.

Demonstrating a pathological role for excess iron in promoting renal injury, iron chelation or dietary restriction reduces proteinuria in rodent models of renal fibrosis, minimal change disease, diabetes and chronic kidney disease (24–29). A small-scale study of patients with glomerulonephritis or diabetic nephropathy also reported reduced proteinuria following iron chelation (30). Implicating iron in the pathogenesis of renal injury in SLE, treating female NZBWF1 mice with the iron chelator deferiprone delayed the onset of albuminuria (14). Treatment of female MRL/*lpr* mice with recombinant hepcidin reduced renal iron accumulation, inflammation and injury (21). In contrast, dietary interventions to induce iron deficiency or to supplement dietary iron intake well above normal levels produced corresponding decreases and increases, respectively, in kidney iron levels in female MRL/*lpr* mice, but in both cases accelerated the development of proteinuria, worsened histological evidence of renal damage and accelerated mortality (31). A recent cross-sectional study of Mexican-mestizo SLE patients noted an association between increased disease activity, excess weight, as well as a high prevalence of nutritional deficiencies, including of iron (32). The mechanisms underlying the pathological effects of iron deficiency or iron excess on disease severity and renal injury specifically in SLE remain to be investigated but likely reflect a combination of direct effects at the tissue level, as well as on the immune system. Data in the current study and a recent study by Scindia and colleagues (21) suggest that, like in female NZBWF1 mice, increased renal iron accumulation occurs in MRL/*lpr* mice as albuminuria develops, even when maintained on a normal diet. Accordingly, we speculate that the use of iron chelators, dietary intervention, or treatments leveraging the iron homeostatic regulatory properties of hepcidin to normalize renal iron levels may provide a direct renoprotective effect in SLE. However, care would need to be taken to avoid creating systemic iron deficiency, which may exacerbate disease activity.

Our findings of increased urinary transferrin excretion once albuminuria has developed in the MRL/*lpr* model in the current study and in NZBWF1 mice previously (14) are congruent with clinical reports of increased urinary transferrin excretion in lupus nephritis patients (5, 7, 17, 18). Whether changes in urinary transferrin filtration and excretion represent an epiphenomenon or an accomplice to renal injury in lupus nephritis remains a tantalizing question. A prospective study reported continued high urinary transferrin levels in pediatric SLE patients who displayed renal function decline within 12 months of biopsy (33). The same group reported that marked reduction of urinary transferrin during treatment is predictive of achieving remission (34). Conversely, a prospective cross-sectional study of patients with primary glomerulonephritis, diabetic nephropathy and other conditions did not find a correlation between urinary transferrin and rate of change in estimated creatinine clearance (35), suggesting that further study is needed to determine the impact of an increased filtered load of transferrin on renal function and parenchymal injury in lupus and other forms of proteinuric renal disease. Our previously published data (14) showed dramatically increased urinary transferrin excretion in female NZBWF1 mice around the onset of albuminuria, and increased renal accumulation of ^59^Fe-transferrin following acute i.v. injection in albuminuric NZBWF1 mice. This suggests that enhanced glomerular filtration of transferrin, which is close to the size exclusion limit of the healthy filtration barrier, occurs once the glomerular filtration barrier is compromised, and then some of the filtered transferrin is then reabsorbed by the kidney. Renal iron deposition in proximal tubular cells has been described in diabetic models (36), and tubulointerstitial fibrosis was ameliorated by iron chelation (28). Proximal tubular iron deposition was also reported in biopsy samples of patients with a variety of chronic renal diseases, with nephrotic patients showing greater amounts of lysosomal iron than non-nephrotic patients, and damaged tubules containing more iron than tubules with less damage (23). Uptake of filtered transferrin across the apical membrane of tubular epithelial cell occurs not only via the transferrin receptor 1 but also via the megalin–cubilin system (37–39). Regulation of expression of these molecules differs, with transferrin receptor 1 expression homeostatically-regulated by cellular iron levels; under conditions of iron overload, transferrin receptor 1 is downregulated while megalin–cubilin expression increases (40). We therefore speculate that the megalin–cubilin system might constitute a relatively unconstrained pathway under pathological conditions. The current study extends these apparent findings of increased nephron exposure to filtered transferrin to male MRL/*lpr* mice, offering an additional model in which to study the mechanistic impact of dysregulated renal transferrin handling in SLE.

Expression of the iron storage protein H-ferritin was dramatically increased in the kidneys of the albuminuric MRL/*lpr* compared to MRL/MpJ mice. This finding complements our previously published observations in female NZBWF1 mice at 34 weeks of age, approximating the median age of onset of albuminuria, wherein ferritin levels were approximately doubled in proximal tubules, medullary thick ascending limbs and distal tubules compared with age-matched NZW mice (14). On the one hand, increased expression of ferritin in both models of SLE would serve as a homeostatic response to safely store excess cellular iron. However, it could also serve as a future liability by providing substrate for autophagic turnover of ferritin and release of catalytic iron, a process that promotes ferroptosis (41). The potential role of ferroptosis in mediating renal damage in SLE awaits further study; given evidence of impaired anti-oxidant defenses, including reduced levels of the key anti-ferroptotic enzyme glutathione peroxidase 4 in the kidneys of MRL/*lpr* mice (42), this seems an intriguing avenue of investigation. Additionally, ferritin is known to be an acute-phase reactant and regulated by proinflammatory cytokines, raising questions regarding the impact of interactions between the inflammatory milieu and renal iron homeostasis as disease progresses.

In summary, this study reports increased renal non-heme iron levels and H-ferritin expression in albuminuric male MRL/*lpr* mice, in association with an increase in urinary transferrin excretion. These data are consistent with previous findings in female NZBWF1 mice. While gathering equivalent renal tissue data from human subjects poses a challenge, increased urinary transferrin represents a commonality, supporting the potential translational relevance of these findings and suggesting that dysregulation of renal iron homeostasis may be a feature of active lupus nephritis.

## Data Availability Statement

The raw data supporting the conclusions of this article will be made available by the authors, without undue reservation.

## Ethics Statement

The animal study was reviewed and approved by Institutional Animal Care and Use Committee, University of Nebraska Medical Center.

## Author Contributions

All authors conducted experiments, participated in data acquisition and analysis, and editing of the manuscript. EB designed the experiments and drafted the manuscript.

## Conflict of Interest

The authors declare that the research was conducted in the absence of any commercial or financial relationships that could be construed as a potential conflict of interest.
